# Molecularly engineered hole-transport material for low-cost perovskite solar cells†

**DOI:** 10.1039/c9sc05694g

**Published:** 2020-01-13

**Authors:** Babak Pashaei, Sebastiano Bellani, Hashem Shahroosvand, Francesco Bonaccorso

**Affiliations:** Group for Molecular Engineering of Advanced Functional Materials (GMA), Chemistry Department, University of Zanjan Zanjan Iran Shahroos@znu.ac.ir; Graphene Labs, Istituto Italiano di Tecnologia via Morego 30 16163 Genova Italy Francesco.bonaccorso@iit.it; BeDimensional SpA Via Albisola 121 16163 Genova Italy

## Abstract

Triphenylamine-*N*-phenyl-4-(phenyldiazenyl)aniline (TPA-AZO) is synthesized *via* a facile CuI-catalyzed reaction and used as a hole transport material (HTM) in perovskite solar cells (PSCs), as an alternative to the expensive spiro-type molecular materials, including commercial 2,2′,7,7′-tetrakis[*N*,*N*-di(4-methoxyphenyl)amino]-9,9′-spirobifluorene (spiro-OMeTAD). Experimental and computational investigations reveal that the highest occupied molecular orbital (HOMO) level of TPA-AZO is deeper than that of spiro-OMeTAD, and optimally matches with the conduction band of the perovskite light absorber. The use of TPA-AZO as a HTM results in PSC prototypes with a power conversion efficiency (PCE) approaching that of the spiro-OMeTAD-based reference device (17.86% *vs.* 19.07%). Moreover, the use of inexpensive starting reagents for the synthesis of TPA-AZO makes the latter a new affordable HTM for PSCs. In particular, the cost of 1 g of TPA-AZO ($22.76) is significantly lower compared to that of spiro-OMeTAD ($170–475). Overall, TPA-AZO-based HTMs are promising candidates for the implementation of viable PSCs in large-scale production.

## Abstract 2

Organic hole transport materials (HTMs) strongly affect the cost of efficient perovskite solar cells. In this work, a newly engineered HTM based on triphenylamine is proposed as a cheap alternative to efficient organic HTMs (*e.g.*, spiro-OMeTAD).

## Graphical abstract

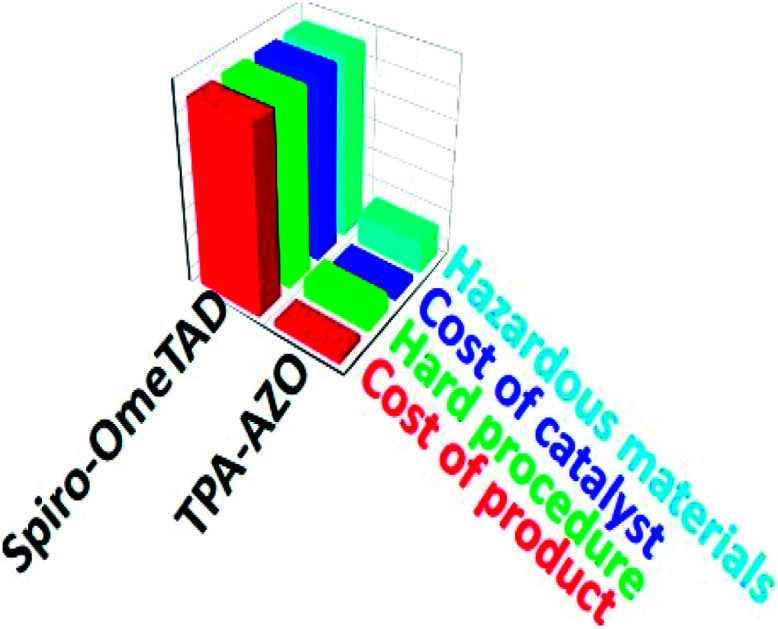

## Introduction

During recent years, the growth of power conversion efficiency (PCE) in perovskite solar cells (PSCs) has stunned the photovoltaic community.^1^ From 2008 to 2019, the record-high PCE of PSCs has increased from 3.8% (ref. 2) to beyond 25.2%,^3^ which is an unprecedented trend in the history of photovoltaics. To boost the PCE of PSCs, the perovskite photoactive layer must work synergistically with the other functional components of the cell, such as the charge transport layer (CTL) and the current collector.^4^ In particular, the photo-generated charges have to be extracted by the perovskite absorber and directed towards the current collector through the CTL.^5^ Consequently, the appropriate tailoring of energy levels at the interfaces is of paramount importance to eliminate/reduce the energy barrier and/or the interfacial structural/morphological defects, which are the cause of charge losses,^6^ hysteresis phenomena^7^ and instability effects.^8^ While several effective electron transport materials (ETMs) are available,^9^ including metal oxides^10^ (*e.g.*, TiO_2_,^11^ SnO_2_,^12^ ZnO,^13^ ZrO_2_ (ref. 14)), metal chalcogenides^15^ and organic materials^16^ (*e.g.*, fullerene^17^ and graphene^18^), the development of efficient, stable and low-cost hole transport materials (HTMs) is a major challenge for the further progress of the PSC technology.^19^

Two different categories of HTMs, namely organic^20^ and inorganic materials,^21^ have been extensively investigated and introduced into PSCs. Inorganic HTMs are especially promising because of their low intrinsic chemical stability and moderate costs,^22^ directly competing with the so-called carbon-based PSCs (*i.e.*, PSCs based on carbon HTMs and/or carbon electrodes).^23^ In fact, efficient PSCs based on inorganic HTMs including CuI,^24^ CuSCN,^25^ CoO_*x*_,^26^ NiO_*x*_,^27^ Cu_2_ZnSnS_4_,^28^ CuCrO_2_,^29^ V_2_O_5_,^30^ MoO_*x*_ and WO_*x*_,^31^ CuAlO_2_,^32^ CuGaO_2_,^33^ CuS^34^ and CuO_*x*_^35^ have been successfully reported. Typically, these HTMs do not require the use of dopants, which can be the source of degradation effects and additional costs. Meanwhile, organic molecular HTMs, such as 2,2′,7,7′-tetrakis[*N*,*N*-di(4-methoxyphenyl)amino]-9,9′-spirobifluorene (spiro-OMeTAD) and poly[bis(4-phenyl)(2,4,6-trimethylphenyl)amine] (PTAA), have been established in PSCs with record-breaking PCEs,^36^ which are typically superior to those achieved using inorganic HTMs.^22*a*,*b*^ However, these HTMs have both technical and economic issues. In fact, on one hand, they often need hygroscopic dopants (such as bis(trifluoromethylsulphonyl)imide (LiTFSI) and 4-*tert*-butylpyridine (TBP), as well as Co(iii) complexes) that trigger degradation of the perovskite layer.^37^ On the other hand, their high cost represents a fundamental hurdle to scale up the manufacturing of PSCs. For example, the cost of spiro-OMeTAD is between 170 and 475$ g^−1^,^38^ contributing to ∼10% of the overall cost of perovskite solar modules.^39^ The PTAA cost is more than twice that of spiro-OMeTAD.^40^ The high cost of organic HTMs is mainly related to their production *via* multi-step synthetic routes, as well as several purification rounds, which need expensive starting reagents and/or catalysts. Actually, the synthesis of these HTMs involves the Pd-catalyzed amination of aryl halides,^41^ which is significantly more complex compared to the Heck,^42^ Stille,^43^ and Suzuki^44^ reactions frequently used in the synthesis of organic molecules.^45^ For example, prototypical HTMs based on spiro-organic derivatives are typically synthesized *via* Pd-catalyzed cross-coupling reactions, mainly contributing to the final cost of the materials. Therefore, tremendous effort has been directed to developing new molecularly engineered organic HTMs through inexpensive synthetic protocols.^46^ To the best of our knowledge, two of the cheapest HTMs reported in the literature are the EDOT-OMeTPA (a small molecule based on triphenylamine (TPA) and 3,4-ethylenedioxythiophene (EDOT) moieties) (∼$10 g^−1^)^38^ and the spiro[fluorene-9,9′-xanthene] (SFX) derivatives (∼$16 g^−1^).^47^ Unfortunately, PSCs based on these HTMS have reached PCEs inferior to 13%, pointing out the need to further optimize such HTMs to reach the state-of-the-art performance of PSCs. Recently, poly(3-hexylthiophene) (P3HT) has also been used as a dopant-free organic HTM, allowing PSCs to reach a certified PCE of 22.7%. This impressive result was achieved using a new device architecture based on a wide-bandgap halide perovskite formed on top of a narrow-bandgap light-absorbing layer by an *in situ* reaction of *n*-hexyl trimethyl ammonium bromide on the perovskite surface.^36*e*^ This strategy was the key to significantly increase the PCE of PSCs based on P3HT as HTMs (typically between 16% and 18% for doped P3HT,^48^ and lower than 15% for pristine P3HT^48*a*^). It is noteworthy that advanced architectures, showing superior stability and/or PCEs, have also been achieved by coupling organic HTMs with inorganic interlayers, such as transition metal dichalcogenides (*e.g.*, MoS_2_),^5*b*,49^ indicating the possibility of improving the performance of PSCs currently fabricated using organic HTMs.

Among the viable organic HTMs, TPA derivatives represent promising candidates to replace the aforementioned expensive benchmarking organic materials. Beyond their facile and cheap synthesis, their TPA moiety is an electron donor unit,^50^ showing the ability to transport positive charges efficiently.^51^ Moreover, their low ionization potential, which results from the amine nitrogen atom,^51*b*^ can minimize the hole oxidizability injection barrier from the transparent anodes used in optoelectronic devices.^52^ Experimental results have proven that TPA derivatives display chemical and thermal stabilities.^53^ Lastly, their high solubility in organic solvents allows them to be processed through established printing/coating techniques.^53^ Not by a chance, the TPA unit has been effectively investigated in various optoelectronic materials used for PSCs,^54^ dye-sensitized solar cells (DSSCs),^55^ organic light-emitting diodes (OLEDs),^56^ and organic field-effect transistors (OFETs).^57^

The TPA molecule also represents a versatile platform for the incorporation of different substituents at different positions. Therefore, it can be an ideal building block for the construction of HTMs based on pseudo-three dimensional conjugated architectures, whose tunable energy levels can efficiently collect/block the photogenerated holes/electrons from the perovskite layer.^58^ In this context, aromatic AZO compounds, which include an Ar–N=N–Ar moiety (in which Ar is an aromatic ring and the –N=N– is the AZO functional group), represent interesting functional groups for the TPA unit. In fact, they display distinctive photochemical and photophysical properties, including chemical and thermal stability, as well as their manifestation in two *cis*–*trans* isomeric forms, which easily and reversibly convert into one another.^59^ Consequently, aromatic AZO compounds have been already exploited in the chemical industry as dyes/pigments,^60^ therapeutic agents,^61^ radical reaction initiators,^62^ drug delivery systems,^63^ nonlinear optics,^64^ photochemical molecular switches,^65^ molecular shuttles,^66^ nanotubes,^67^ and eye glasses and optical filters.^68^ Specifically for PSCs, the *cis*–*trans* isomerisation of aromatic AZO derivatives could be exploited to modulate the hole mobility of TPA-based HTMs.^69^ However, the combination of TPA and AZO into a new HTM for PSCs has not been reported yet.

Herein, we report the synthesis of aromatic AZO-functionalized TPA (TPA-AZO) to be investigated as a possible low-cost HTM in PSCs. Our results show the possibility to achieve PCEs approaching those of benchmarking devices based on organic HTMs, but in a cost-effective way.

## Results and discussion

**Fig. 1:**
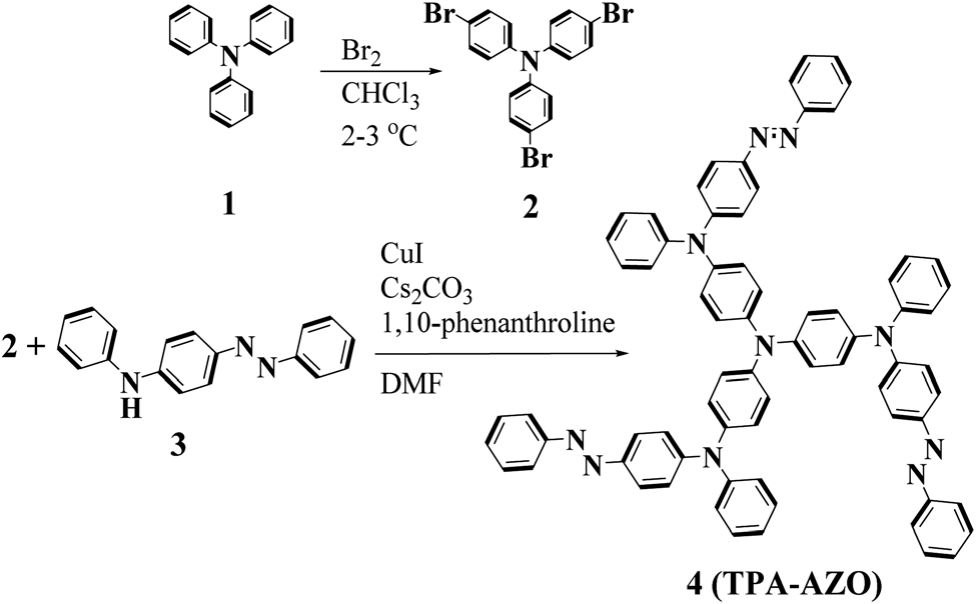
Synthesis reactions and molecular structure of TPA-AZO.

**Fig. 2:**
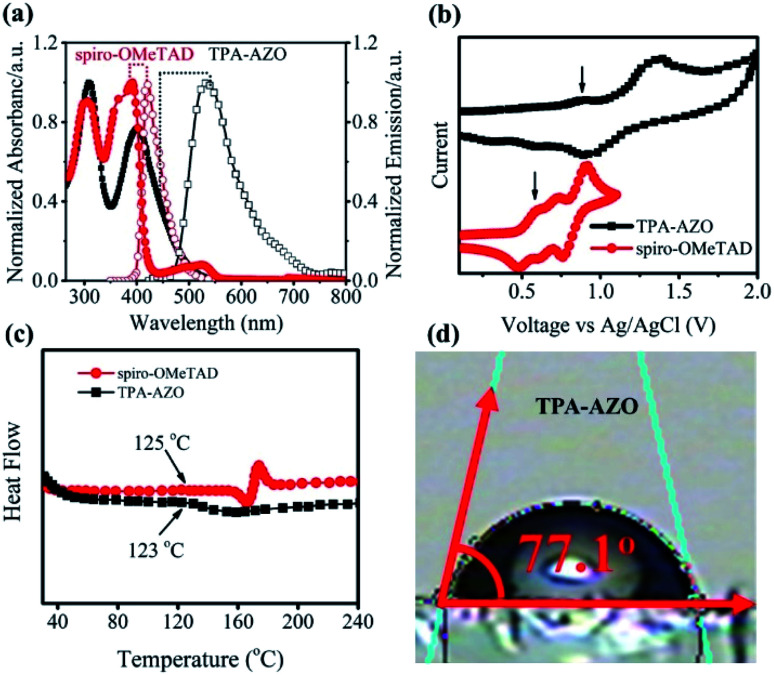
(a) UV-vis absorption and PL emission spectra of TPA-AZO and spiro-OMeTAD. (b) CV curves of spiro-OMeTAD and TPA-AZO. (c) DSC curves of TPA-AZO and spiro-OMeTAD. (d) Contact angles of TPA-AZO.

The synthetic route and molecular structure of TPA-AZO are shown in Fig. 1, while the procedure details are reported in the Experimental section. Briefly, TPA-AZO was synthesized through a two-step reaction, including: (1) the bromination of TPA in CHCl_3_ solution^70^ (see ESI, Fig. S1†) and (2) the amination reaction between tribromotriarylamine (t-Br-TPA) and *N*-phenyl-4-(phenyldiazenyl)aniline. All the compounds were purified through recrystallization and column chromatography. TPA-AZO was characterized by spectroscopic, electrochemical and thermoanalytical techniques, and the results were compared with those measured for spiro-OMeTAD, hereafter assumed as the HTM benchmark. The absence of the singlet at 6.07 ppm in the ^1^HNMR spectra of TPA-AZO (Fig. S2†), which is related to N–H of *N*-phenyl-4-(phenyldiazenyl)aniline, confirms the successful synthesis of TPA-AZO. Fig. 2a shows the ultraviolet-visible (UV-vis) absorption and photoluminescence (PL) spectra of the investigated HTMs.

The UV-vis absorption spectra of TPA-AZO and spiro-OMeTAD exhibit two main bands attributed to π → π* and n → π* transitions.^71^ The second band of TPA-AZO is red-shifted compared to that of spiro-OMeTAD. The PL emission of TPA-AZO is also red-shifted (by 115 nm) relative to that of spiro-OMeTAD. The optical band gap (*E*_o–o_) of the materials can be qualitatively estimated by the energy corresponding to the intersection between the UV-vis absorption and the PL spectra. Therefore, the *E*_o–o_ of TPA-AZO is smaller than that of spiro-OMeTAD. However, the intensity of the n → π* band in the spectrum of TPA-AZO is lower than the corresponding one of spiro-OMeTAD. This indicates that the synthesized TPA-AZO can minimize light absorption in the visible spectral region. Thus, TPA-AZO can in principle reduce the absorption of visible light reflected by the back contact, as well as it can be effectively used in bifacial PSCs.^59,66^

Cyclic voltammetry (CV) analysis was carried out to determine the highest occupied molecular orbital (HOMO) levels of TPA-AZO and spiro-OMeTAD (Fig. 2b).^72^ The CV curve of TPA-AZO shows two reversible oxidation peaks, while the CV curve of spiro-OMeTAD exhibits three reversible oxidation peaks, in agreement with previous studies.^73^ Notably, the oxidation/reduction potentials of TPA-AZO are higher than those of spiro-OMeTAD (data are summarized in Table 1). The estimated values for the HOMO energies (*E*_HOMO_) of TPA-AZO and spiro-OMeTAD are −5.39 eV and −5.20 eV, respectively (according to the equation: *E*_HOMO_ = −(*E*_ox_ (*vs.* Fc/Fc^+^) + 4.8 eV)). The lowest unoccupied molecular orbital (LUMO) energies (*E*_LUMO_) of the materials, as calculated by *E*_LUMO_ = *E*_HOMO_ + *E*_o–o_, are −2.55 eV and −2.16 eV for TPA-AZO and spiro-OMeTAD, respectively.

**Table 1:** Optical and electrochemical data for TPA-AZO and spiro-OMeTAD HTMs

HTM	*λ*_abs_ a [nm]	*λ*_em_ a [nm]	*E*_o–o_ b [eV]	*E*_OX_ c [V]	*E*_HOMO_ d [eV]	*E*_LUMO_ e [eV]	*T*_g_ [°C]	*η*_quench_	Hole mobility (pristine state) [cm^2^ V^−1^ s^−1^]	Hole mobility (doped state) [cm^2^ V^−1^ s^−1^]
TPA-AZO	308, 401	535	2.84	0.79	−5.39	−2.55	123	0.83	9.4 × 10^−5^	1.2 × 10^−4^
Spiro-OMeTAD	304, 389	421	3.04	0.6	−5.20	−2.16	124	0.94	2.6 × 10^−5^	2.0 × 10^−4^

a UV-Vis and photoluminescence spectra were measured in CHCl_3_ solution.

b *E*_o–o_ was estimated by the energy corresponding to the intersection of the UV-Vis absorption and PL spectra.

c From CV measurements, *E*_1/2_ = 1/2(*E*_pa_ + *E*_pc_); 0.1 M chloroform/tetrabutylammonium perchlorate (TBAP) *versus* Ag/AgCl at scan rate of 80 mV s^−1^.

d *E*_HOMO_ = −(*E*_ox_ (*vs.* Fc/Fc^+^) + 4.8 eV).

e *E*_LUMO_ = *E*_HOMO_ + *E*_o–o_.

**Fig. 3:**
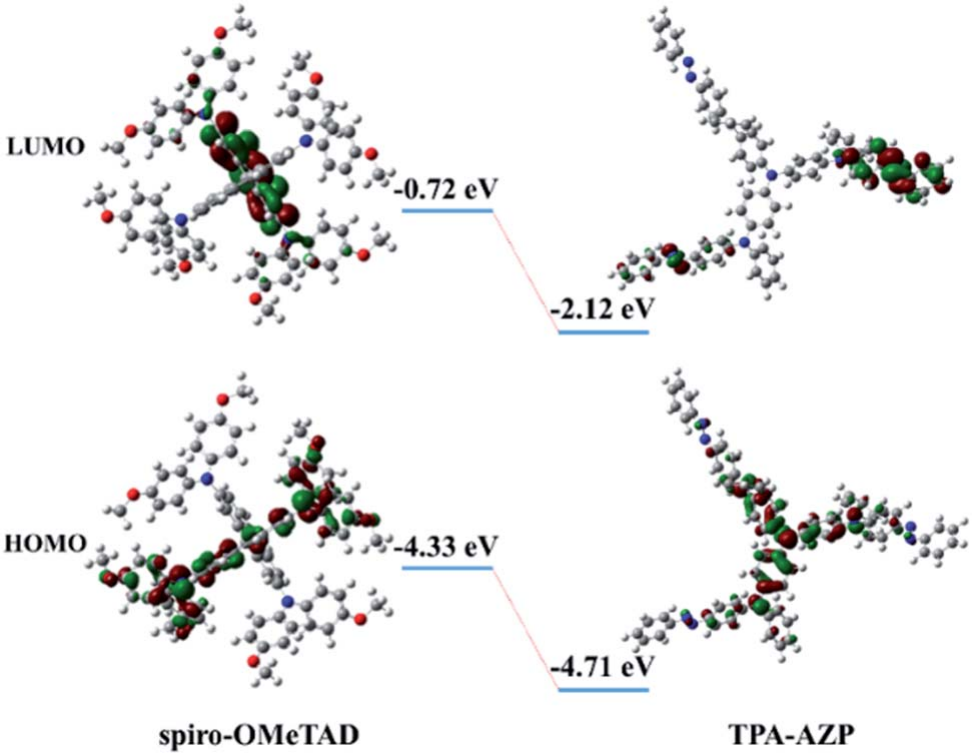
Calculated frontier molecular orbitals of TPA-AZO.

Density functional theory (DFT) calculations for the energy levels of TPA-AZO and spiro-OMeTAD (Fig. 3) also indicate that the HOMO level of TPA-AZO is deeper than that of spiro-OMeTAD, evidencing its optimal matching with the HOMO level of the perovskite layer (−5.43 eV).^38^

Differential scanning calorimetry (DSC) analysis was carried out to investigate the thermal stability of TPA-AZO, which was compared with that of spiro-OMeTAD (Fig. 2c). The DSC curves indicate that TPA-AZO has a glass transition temperature (*T*_g_) of about 123 °C, which is very close to the *T*_g_ of spiro-OMeTAD (124 °C).^74^ These results indicate that TPA-AZO shows a similar thermal stability behaviour to spiro-OMeTAD, which undergoes a severe morphological deformation at temperature higher than 80 °C.^75^ One of the main strategies to enhance the stability of PSCs is the use of hydrophobic HTMs to prevent the diffusion of water and moisture into the photoactive layer of the cells.^76^ In fact, hydrophobic HTMs can act as barrier layers to avoid the water-/moisture-induced decomposition of CH_3_NH_3_PbI_3_ to CH_3_NH_3_I and PbI_2_.^77^ Therefore, water contact angle measurements were carried out on the TPA-AZO surface to examine its hydrophobicity. As shown in Fig. 2d, the measured water contact angle for TPA-AZO is 77°. This result indicates that TPA-AZO is hydrophobic and might prevent the penetration of water into the perovskite layer, thus limiting degradation effects.^76*c*^

**Fig. 4:**
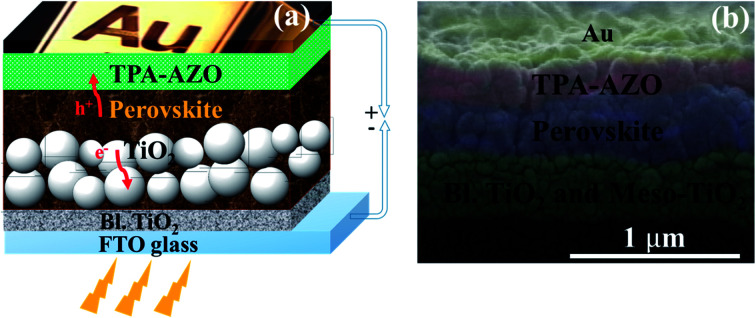
(a) Sketch of the mesoscopic architecture adopted for the investigated PSCs. (b) SEM cross section of a representative PSC based on the TPA-AZO HTM.

As suggested by its spectroscopic, electrochemical and analytical characterization, TPA-AZO was subsequently used as a HTM in PSCs. The cells were fabricated by using the following mesoscopic architecture:^37*b*^ glass-FTO/TiO_2_ (compact layer)/TiO_2_ (mesoporous layer)/(FAPbI_3_)_0.85_(MAPbBr_3_)_0.15_/TPA-AZO or spiro-OMeTAD/Au (Fig. 4). The layered structure of the PSC architecture was confirmed by scanning electron microscopy (SEM) (Fig. 4b).

**Fig. 5:**
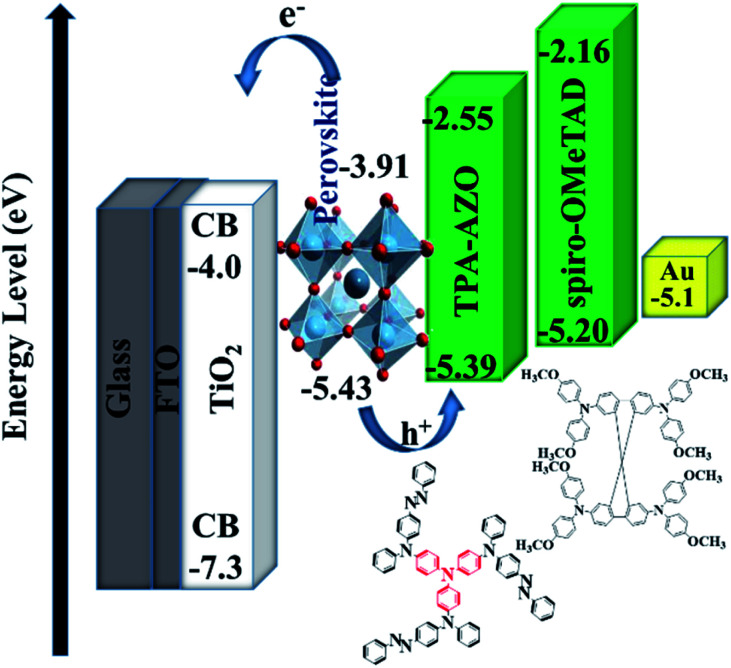
Energy level diagram of the corresponding materials used in the investigated PSCs.

The energy level diagram in Fig. 5 shows that the estimated HOMO level of TPA-AZO optimally matches that of the perovskite. In particular, the energy difference between the HOMO levels of the perovskite and TPA-AZO (ΔHOMO_perovskite–HTM_) ( ∼0.04 eV) is lower than that between the HOMO levels of the perovskite and spiro-OMeTAD (∼0.23 eV). This means that TPA-AZO reduces the energy barrier related to hole extraction relative to the case of spiro-OMeTAD.

Effective charge carrier transport is another important feature determining the design of efficient HTMs.^78^ The hole mobilities of the HTMs were investigated by means of the space-charge limited current (SCLC) method, in agreement with previous literature.^79^ Thus, the mobility values were extracted from the *J*–*V* curves (Fig. S3†) measured for each HTM. The estimated data are reported in Table 1 for both TPA-AZO and spiro-OMeTAD. The estimated hole mobility of pristine TPA-AZO is 9.8 × 10^−4^ cm^2^ V^−1^ s^−1^, which is more than one order of magnitude higher than that of spiro-OMeTAD (2.6 × 10^−5^ cm^2^ V^−1^ s^−1^). After doping the HTMs with LiTFSI and TBP, the hole mobility increases compared to that of the pristine samples, reaching values of 1.2 × 10^−4^ cm^2^ V^−1^ s^−1^ and 2.0 × 10^−4^ cm^2^ V^−1^ s^−1^ for doped TPA-AZO and doped spiro-OMeTAD, respectively. However, the hole mobility data and the conductivity values measured for the pristine HTMs are useful information. In fact, the elimination of the use of dopant would increase the stability of the PSCs, while reducing their overall cost.^80^

**Fig. 6:**
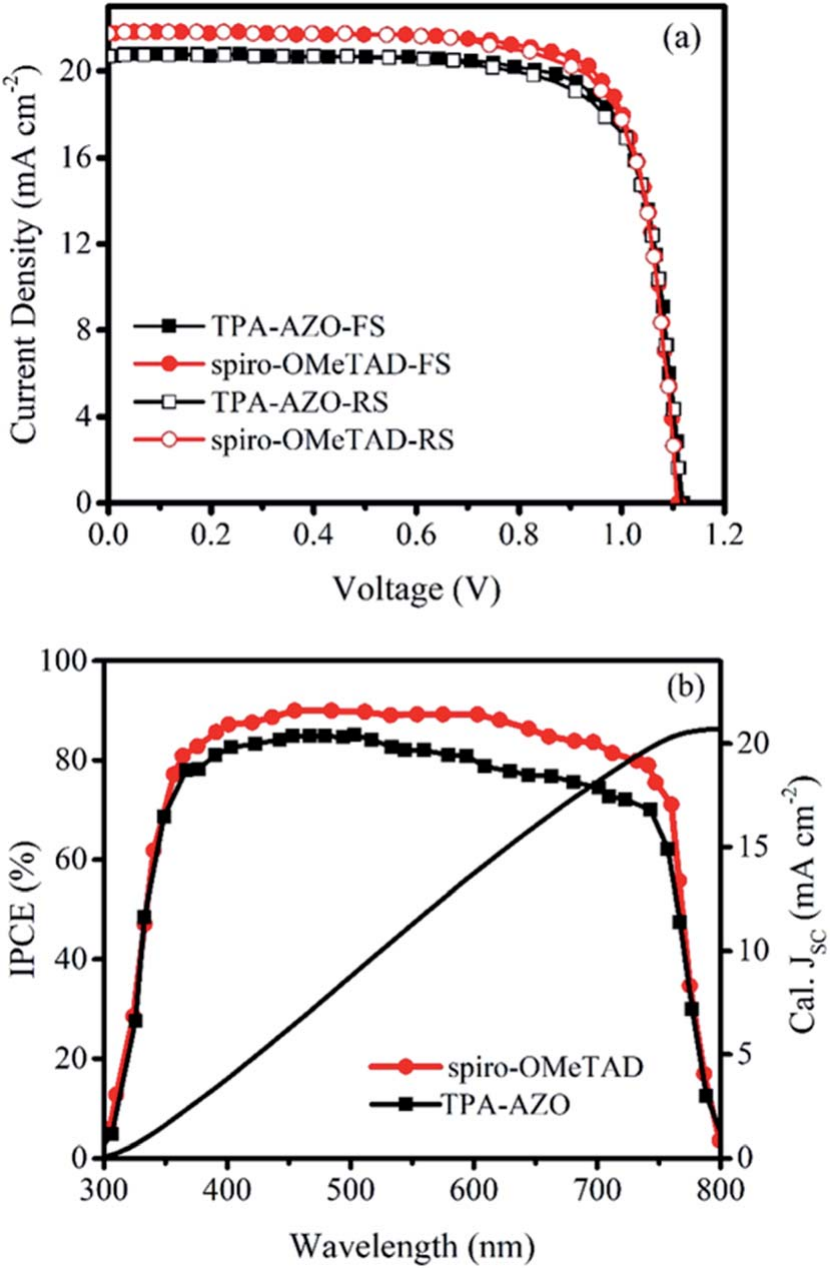
(a) *J*–*V* and (b) IPCE curves of the PSCs based on TPA-AZO and spiro-OMeTAD.

The photovoltaic performances of the PSCs based on the doped TPA-AZO and spiro-OMeTAD were investigated by measuring their current density–voltage (*J*–*V*) curves under AM1.5G illumination (Fig. 6a). The corresponding photovoltaic parameters are summarized in Table 2, which also reports the data obtained for the PSCs using the dopant-free HTMs, whose *J*–*V* curves are reported in Fig. S4.†

**Table 2:** Summary of the figures of merit of the PSCs based on TPA-AZO and spiro-OMeTAD under AM1.5G illumination

HTM	*J*_SC_ [mA cm^−2^]	*V*_OC_ [V]	FF [%]	PCE [%]
Dopant-free TPA-AZO	17.01	0.94	63	10.07
TPA-AZO-forward scan (FS)	20.72	1.12	77	17.86
TPA-AZO-reverse scan (RS)	20.67	1.12	76	17.59
Dopant-free spiro-OMeTAD	14.69	0.79	47	5.45
Spiro-OMeTAD-FS	21.75	1.11	79	19.07
Spiro-OMeTAD-RS	21.73	1.11	77	18.57

The PSC based on TPA-AZO shows a short-circuit current density (*J*_SC_) of 20.72 mA cm^−2^, an open-circuit voltage (*V*_OC_) of 1.12 V, and a fill factor (FF) of 0.77, leading to a PCE of 17.86% (forward scan –FS–). This PCE value is inferior to that measured for the reference PSC, *i.e.*, the spiro-OMeTAD one, PCE = 19.07% (FS). However, the PCE achieved by using TPA-AZO is significantly superior to those previously achieved by using low-cost organic HTMs in conventional architectures (*e.g.*, 11% for EDOT-OMeTPA^38^ and 12.4% for SFX derivatives^47^). As shown in Fig. 6a, the photovoltaic parameters obtained by measuring *J*–*V* curves in reverse scan (RS) mode were similar to those measured with FS. The statistical analyses of the photovoltaic parameters measured for 30 samples of each doped HTM-based PSC are reported in Fig. S4,† showing average PCEs of 17.17% and 15.93% for the PSCs based on spiro-OMeTAD and TPA-AZO, respectively. In addition, the PSCs based on dopant-free TPA-AZO yielded a PCE of 10.07%, which is significantly higher than that of dopant-free spiro-OMeTAD (5.45%) (Table 2, Fig. S5†). As shown in Fig. S6,† the dopants do not affect the film morphology of TPA-AZO, while they are needed to homogenize the morphology of spiro-OMeTAD, in agreement with previous studies.^76*c*,81^ These results indicate that our dopant-free TPA-AZO is promising to be used in advanced PSC architecture, as for example shown with P3HT,^36*e*^ for which its doping is instead recommended for conventional architectures.^48*a*^

The difference between the *J*_SC_ of our optimized PSCs is the major reason for the lower PCE of the TPA-AZO-based PSC compared to that of the spiro-OMeTAD-based reference. Fig. 6b shows the incident photon to current efficiency (IPCE) measurements of the investigated PSCs. The integrated current density (calculated from the IPCE data in the 300–800 nm range under AM1.5G conditions) for the TPA-AZO-based PSC is consistent with the *J*–*V* curve data (*i.e.*, *J*_sc, calc._ ∼ *J*_sc_). In particular, the IPCE of the TPA-AZO-based PSC is lower compared to the spiro-OMeTAD-based reference one in the 450–600 nm spectral range.^82^

**Fig. 7:**
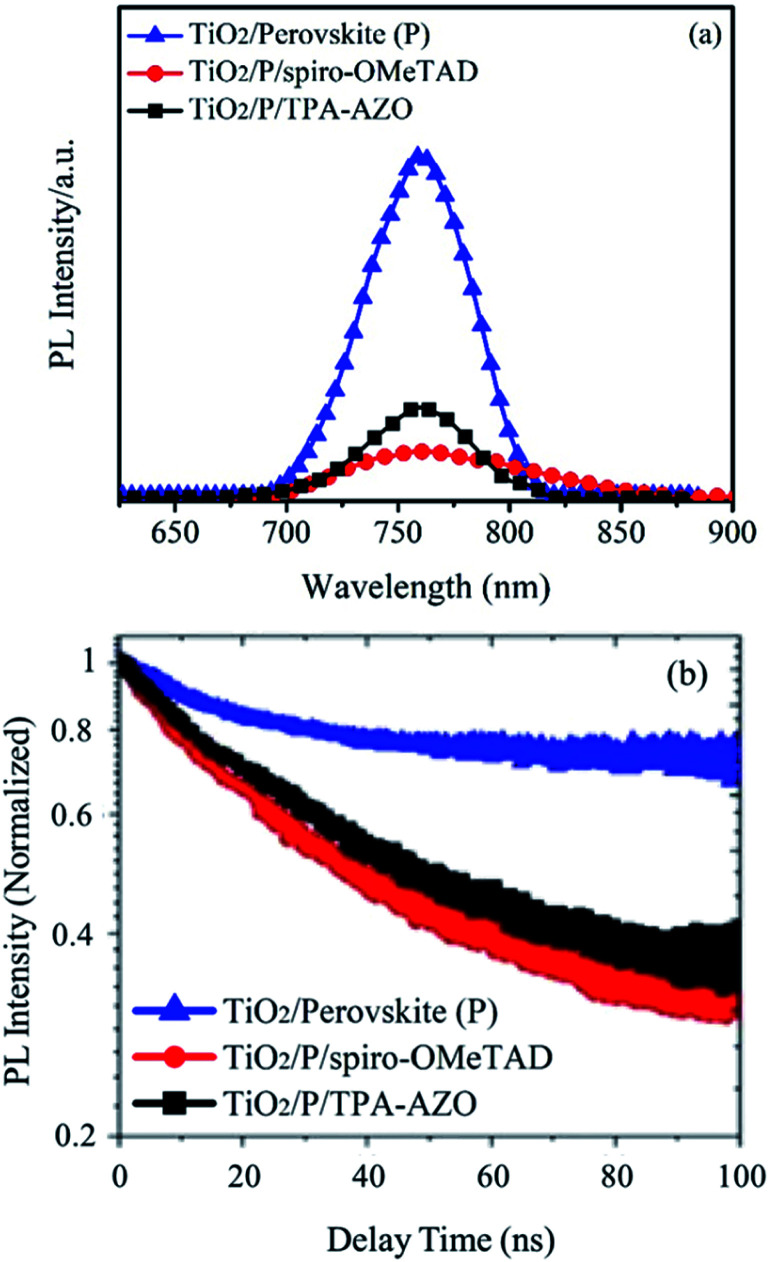
(a) Steady-state PL spectra of the TiO_2_/perovskite, TiO_2_/perovskite/TPA-AZO and TiO_2_/perovskite/spiro-OMeTAD structures. (b) The corresponding PL decay curves measured at a wavelength near the band gap that yields the maximum PL signal upon exciting the perovskite, TiO_2_/perovskite/TPA-AZO- and TiO_2_/perovskite/spiro-OMeTAD structures at 405 nm.

In order to understand the photocurrent losses, steady-state and time-resolved PL spectroscopy measurements were performed to specifically evaluate the capability of the HTMs to extract the photogenerated holes from the perovskite layer. In fact, the hole extraction process hinders radiative charge recombination in the absorber material,^89,90^ which consequently shows a PL quenching.^91^ As shown by the steady-state PL spectra (Fig. 7a), spiro-OMeTAD quenches the PL of the perovskite more effectively than AZO-TPA.^83^ The calculated PL quenching factor (*η*_quench_) for the spiro-OMeTAD-based structure is ∼10% higher than that of TPA-AZO (see values in Table 1).

Time-resolved PL spectroscopy data (Fig. 7b) indicate that the photo-charge lifetime (*τ*_2_) in the presence of spiro-OMeTAD (7.8 ns, as estimated by fitting the PL decay traces with the bi-exponential decay model) is slightly lower than that in the presence of TPA-AZO (9.2 ns). It is noteworthy that both these values are significantly lower than that measured for the HTM-free structures (16.7 ns),^84^ confirming that both the HTMs effectively quench the PL of the perovskite absorber by extracting the photo-generated holes.^85^ Overall, the PL measurements show that, despite the optimal matching between the HOMO levels of TPA-AZO and the perovskite, spiro-OMeTAD is still more efficient to extract holes from the perovskite compared to TPA-AZO.^86^ Nonetheless, the addition of more electron donating groups such as methoxy groups could prospectively improve the performance of TPA-AZO as the HTM.

**Fig. 8:**
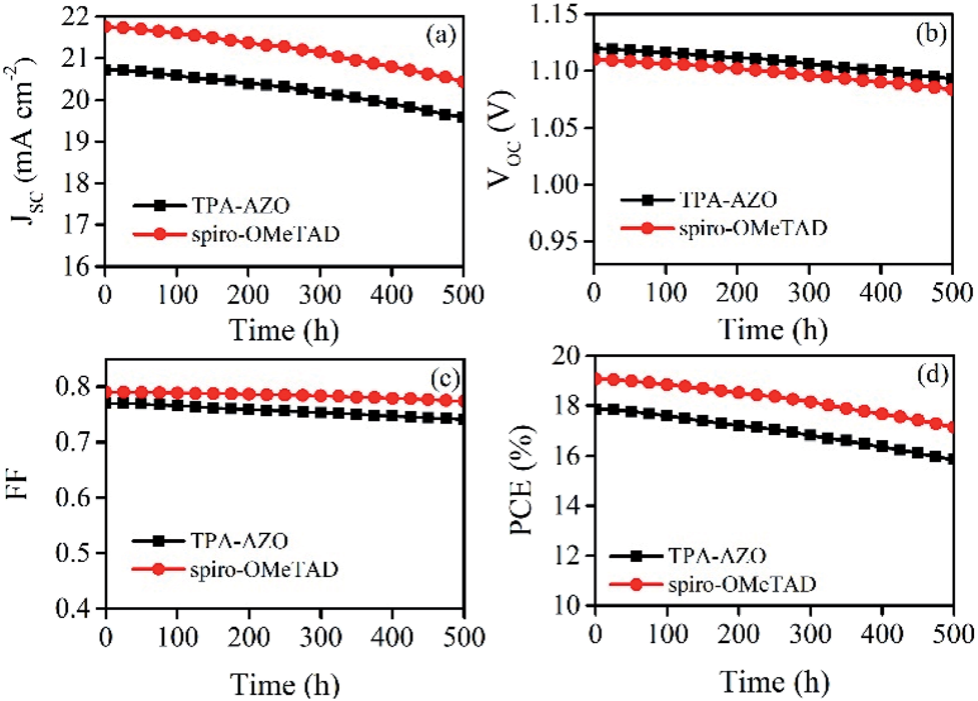
Stability of the photovoltaic characteristics over time for the PSCs based on TPA-AZO and spiro-OMeTAD: (a) *J*_SC_; (b) *V*_OC_; (c) FF; (d) PCE.

The stability of the photovoltaic performance is another crucial aspect of investigation for PSCs, which still suffer from severe degradation over time under realistic operating conditions. As shown in Fig. 8, the photovoltaic performance stability of the PSC based on TPA-AZO HTM is similar to that recorded for the spiro-OMeTAD-based reference. This suggests that degradation effects are attributed to causes not directly ascribed to the HTMs.

In addition, a huge advantage of our newly synthesized TPA-AZO compared with spiro-OMeTAD is related to the preparation costs (much lower for the case of TPA-AZO). In fact, the estimated costs of TPA-AZO and spiro-OMeTAD are $22.76 g^−1^ and $273.62 g^−1^, respectively (Tables S1 and S2†).^38,85,87^ This difference in price is related to the following factors: (1) six experimental steps have to be carried out to produce pure spiro-OMeTAD, while only two steps are needed for TPA-AZO; (2) the catalyst used for the synthesis of TPA-AZO (CuI/Cs_2_CO_3_/phenanthroline) is significantly cheaper than that used for the synthesis of spiro-OMeTAD (Pd/bis(diphenylphosphino)-l,l′-binaphthyl (BINAP)). Noteworthy, the additional costs associated with dopants used for our optimized PSCs are reported in Table S3,† showing that they only marginally contribute to the overall cost of TPA-AZO.

**Fig. 9:**
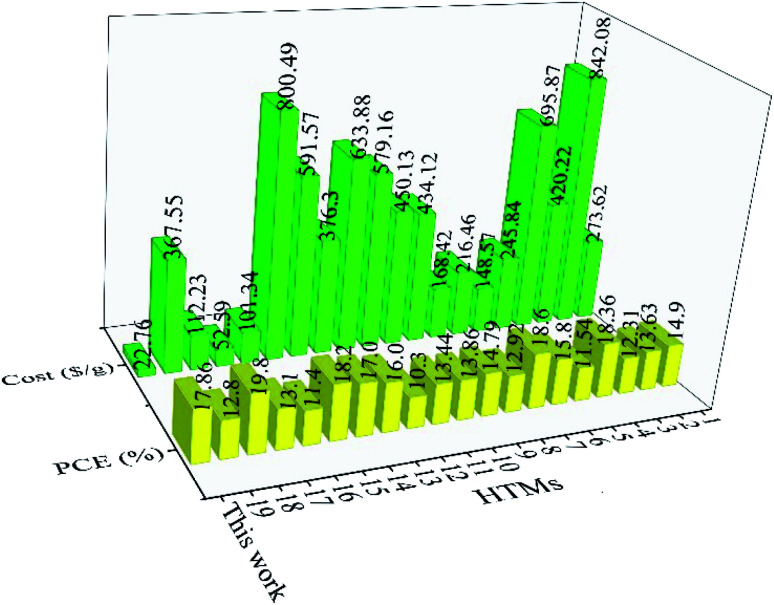
Comparison between costs of organic HTMs (green histograms) and PCEs of the corresponding PSCs (red histograms), as reported in our work and other relevant studies. The HTMs reported in previous literature are named with numbers from 1 to 19. Their full names and schemes are given in ESI, Table S4.†

In addition to spiro-OMeTAD, other small organic HTMs can be compared with our TPA-AZO HTM in terms of synthesis cost and PCE of their corresponding cells (Table S4†).^73,80*a*,88^ For example, the use of star-shaped HTMs, based on TPA with diphenyl ethenyl side arms, resulted in PCEs up to 11.8%.^89^ The use of *p*-methoxy side groups further increased the PCE to 13.63%.^88*g*^ In order to increase their charge transport properties, prevent aggregation and improve their stability, the side arms of these materials were modified with a bis-dimethylfluorenyl amino moiety. The resulting star-shaped structures allowed their PSCs to reach PCE ∼ 18%.^77*d*^ Other interesting examples of organic HTMs based on TPA are bis[(4-methoxyphenyl)aminophenyl]ethene (EtheneDTPA) and tetrakis[(4-me-thoxyphenyl)aminophenyl]ethene (EtheneTTPA), which have the lowest cost for the synthesis of 1 g (101.34 and 52.59$ per g, respectively) among the HTMs reported in Table S4† beyond our TPA-AZO. However, the PCE of their corresponding PSCs is ∼15%, which is lower than those of PSCs based on other low-cost HTMs.^37*b*,38,88*a*,90^ Lastly, various building blocks, such as spirobifluorenes,^91^ thiophenes,^37*b*,38,80*a*,92^ triazatruxenes,^73,93^ azulenes^94^ and other small organic HTMs,^4*a*,88*e*,95^ have been used as the core of several HTMs. Despite their remarkable PCE values, all these HTMs have higher synthesis costs compared to that of TPA-AZO. Thus, our TPA-AZO is still overall advantageous compared to the aforementioned organic HTMs, as shown in Fig. 9.

Our TPA-AZO was further compared with other dopant-free inorganic and polymeric HTMs. In spite of worthy properties of dopant-free inorganic HTMs, including thermal and chemical stability towards moisture exposure, they suffer from several issues. For example, the solvent used for their deposition on the perovskite layer can dissolve the perovskite, thereby affecting the stability of the PSCs.^25*b*,96^ In addition, such HTMs can be low band-gap materials with limited optical transparency,^97^ and/or can have expensive deposition processes (*e.g.*, magnetron sputtering).^98^ Lastly, bare polymers can suffer from complex or costly purification procedures, and/or difficult infiltration into nanostructured materials.^96^ As shown in Table S5,† our PSCs based on dopant-free TPA-AZO exhibit PCEs that are superior to those achieved by several dopant-free HTMs, although high-performance inorganic and polymeric HTMs have been demonstrated.^24,25*b*,28,33,99^ Overall, the distinctive balance between low-cost synthesis of our TPA-AZO and the satisfactory PCE of its corresponding PSCs is promising for the realization of high-efficiency and viable PSCs.

## Conclusion

In conclusion, our results shed light on the engineering of low-cost and effective HTMs based on TPA. In particular, our AZO-functionalized TPA (TPA-AZO) is a promising material candidate to replace the spiro-OMeTAD benchmark. The spectroscopic and electrochemical analyses and the DFT calculations support that the HOMO of TPA-AZO is optimally tuned with that of the perovskite layer. The TPA-AZO-based PSCs achieved a PCE as high as 17.86%, close to that of spiro-OMeTAD. Moreover, the cost of 1 g of TPA-AZO is about one-thirty of that of spiro-OMeTAD. This makes our TPA-AZO one of the cheapest effective HTMs for PSCs. Based on our current knowledge, we believe that the commercialization of TPA-AZO-based PSCs could more affordable than that of spiro-OMeTAD-based PSCs.

## Experimental

### Materials

All starting materials were purchased from Aldrich Chemical and Merck Companies and used without further purification.

### Characterization of materials

^1^H NMR spectra were recorded on Bruker Advance 250 MHz spectrometers, locked on deuterated solvents. Chemical shifts were calibrated against tetramethylsilane as an internal standard. UV-vis absorption and PL spectra were measured using an Ultrospec 3100 pro spectrophotometer and AvaSpec-125 spectrophotometer, respectively. The electrochemical studies were accomplished by using a SAMA500 potentiostat electrochemical analyzer in a three-electrode cell configuration. A Pt disk and a Pt wire were used as the working electrode and the counter electrode, respectively. A KCl-saturated Ag/AgCl electrode was used as reference. The CV measurements were performed in chloroform solvent, using 0.1 M TBAP as the supporting electrolyte.

### Synthesis of triphenylamine-*N*-phenyl-4-(phenyldiazenyl)aniline (TPA-AZO)

Tribromotriarylamine (t-Br-TPA) was prepared according to the procedure reported in literature.^70^ Then, a mixture of CuI (0.050 g), 1,10-phenantholine (0.10 g), Cs_2_CO_3_ (2.00 g), t-Br-TPA (0.482 g, 1 mmol), *N*-phenyl-4-(phenyldiazenyl)aniline (0.982 g, 3.6 mmol) and 5 ml of dimethylformamide (DMF) was heated under reflux for 2 days. After cooling to ambient temperature, the product was extracted by using CHCl_3_ and H_2_O. The combined organic phases were dried over anhydrous MgSO_4_ and evaporated. The crude product was purified by column chromatography (CH_2_Cl_2_ :  hexane 4 : 7) (45%). ^1^H NMR (CDCl_3_, 250 MHz): 7.05–7.25 (m, 9H), 7.31–7.49 (m, 5H), 7.86–7.89 (m, 4H). CHN: anal. calcd for C_72_H_54_N_10_ (%): C, 81.643; H, 5.144; N, 13.227. Found (%): C, 81.649; H, 5.15; N, 13.233. ESI-MS: *m*/*z* 1057.10, [M − H]^+^.

### Fabrication of solar cells

Perovskite solar cells were fabricated on fluorine-doped tin oxide (FTO) coated glass substrates. Part of the glass substrate coated with FTO was etched with Zn powder and 2 M HCl solution in ethanol. Subsequently, the substrates were washed with distilled water, detergent, acetone, ethanol and isopropanol. The samples were then treated with an ultraviolet/O_3_ cleaner for 15 min. On these substrates, a solution of HCl and titanium isopropoxide (TTIP) in anhydrous ethanol (a solution of HCl (35 μl) in ethanol (2.53 ml) was added drop by drop to a solution of TTIP (369 μl) in ethanol (2.53 ml)) was deposited by spin-coating at 2000 rpm for 30 s. Subsequently, the substrates were heated at 500 °C for 30 min and cooled down to room temperature. Mesoporous TiO_2_ diluted in ethanol was deposited by spin-coating at 2000 rpm for 10 s to achieve a 300–400 nm-thick layer. Afterward, the substrates were sintered at 500 °C for 30 min. The PbI_2_ solution was coated on the mesoporous TiO_2_ layer for 5 s at 6500 rpm and dried at 70 °C. The mixed perovskite precursor solution was prepared by dissolving PbI_2_ (1.15 M), formamidinium iodide (FAI) (1.10 M), PbBr_2_ (0.2 M), and methylammonium bromide (MABr) (0.2 M) in an anhydrous solvent DMF : dimethyl sulfoxide (DMSO) = 4 : 1 (volume ratio). The perovskite solution was spin-coated in a two-step procedure at 1000 and 6000 rpm for 10 and 30 s, respectively. Next, TPA-AZO or spiro-OMeTAD were deposited by spin-coating their solutions at 4000 rpm for 20 s. The HTM solutions were prepared by dissolving the HTMs in chlorobenzene at a concentration of 78 mM, with the addition of 18 μl LiTFSI (from a stock solution in acetonitrile with a concentration of 1.0 M) and 29 μl *tert*-butyl pyridine (from a stock solution in chlorobenzene with a concentration of 1.0 M). Finally, an 80 nm-thick Au electrode was deposited by thermal evaporation under high vacuum (∼10^−5^ Pa).^37*b*,85^

### Characterization of solar cells

The *J*–*V* curves were measured using a solar simulator (Newport, Oriel Class A, 91195A) with a source meter (Keithley 2420) under 100 mA cm^−2^ illumination (AM 1.5G) and a calibrated Si-reference cell certified by the National Renewable Energy Laboratory (NREL). The *J*–*V* curves of all devices were measured by masking the active area with a metal mask of area 0.096 cm^2^. The SEM apparatus with the MIRA III model (TESCAN Co.) was used for the acquisition of the SEM images.

### Mobility measurements

Charge transport in the HTMs was investigated by the SCLC method reported in the literature.^79*b*^ Experimentally, the FTO coated glass substrates were prepared according to the “Solar cells fabrication” section. After spin-coating the poly(3,4-ethylenedioxythiophene) polystyrene sulfonate (PEDOT:PSS) layer onto the substrates, the HTM films were deposited by spin-coating the HTM solutions in anhydrous chlorobenzene (10 mg ml^−1^) at 2000 rpm for 20 s. Finally, an Au layer was evaporated onto the active layer under high vacuum (∼10^−5^ Pa). The hole mobility was estimated using Mott–Gurney's equation.

### Computational methods

The ground-state geometries were optimized using density functional theory (DFT) with the B3LYP hybrid functional at the basis set level of 6-31G*, and the frontier molecular orbitals were drawn using an isovalue of 0.03 a.u. All the calculations were performed using the Gaussian 09 package in the PowerLeader workstation. The molecular orbitals were visualized using Gauss View 5.0.8.

## Conflicts of interest

There are no conflicts to declare.

## Supplementary Material

SC-011-C9SC05694G-s001
