# Supplementary material for: Molecularly engineered hole-transport material for low-cost perovskite solar cells
Source: Chem Sci. 2020 Jan 13;11(9):2429–39. doi: 10.1039/c9sc05694g (PMC8157471; doi:10.1039/c9sc05694g)
Supplement: SC-011-C9SC05694G-s001 [file SC-011-C9SC05694G-s001.pdf]

Electronic Supplementary Material (ESI) for Chemical Science.
This journal is © The Royal Society of Chemistry 2020

Supplementary Information

Molecularly Engineered Hole-Transport Material for Low-cost Perovskite Solar Cells

Babak Pashaei,[1] Sebastiano Bellani,[2] Hashem Shahroosvand,[1,*] and Francesco Bonaccorso[2,3,*]

1 Group for Molecular Engineering of Advanced Functional Materials (GMA), Chemistry Department, University of Zanjan, Zanjan, Iran † Electronic

2 Graphene Labs, Istituto Italiano di Tecnologia, via Morego 30, 16163 Genova, Italy.

3 BeDimensional SpA, Via Albisola 121, 16163 Genova, Italy

Corresponding authors:
Hashem Shahroosvand: shahroos@znu.ac.ir
Francesco Bonaccorso: francesco.bonaccorso@iit.it

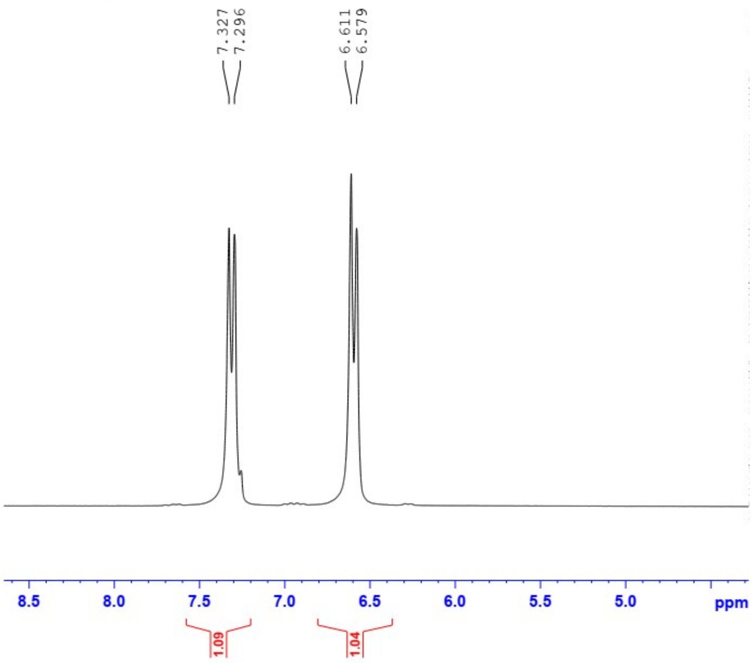

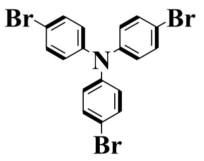

Figure S1. ^{1}H NMR ($CDCl_3$) spectrum of tris(4-bromophenyl)amine (**2**).

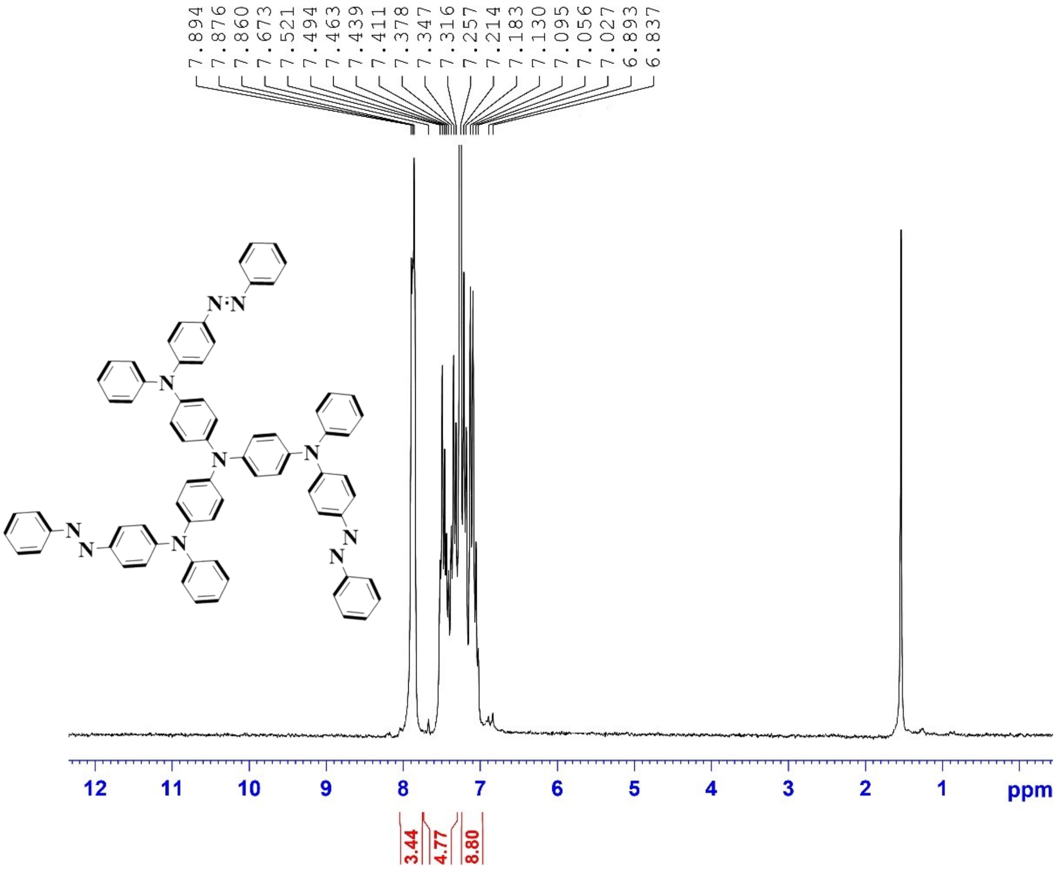

Figure S2. ^{1}H NMR ($CDCl_3$) spectrum of TPA-AZO (**4**).

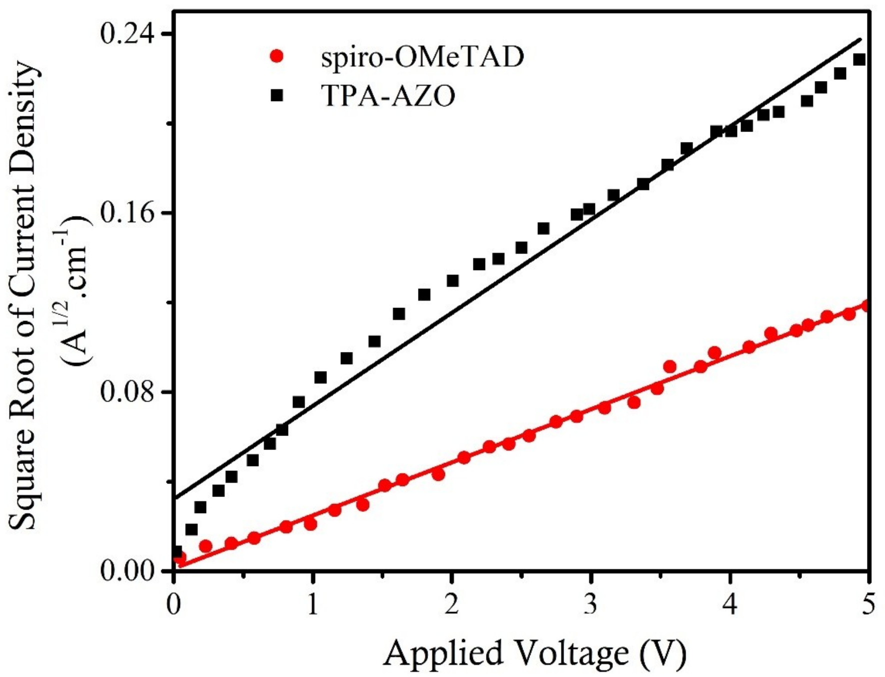

Figure S3. J–V characteristics of thin films of HTM in their pristine state.

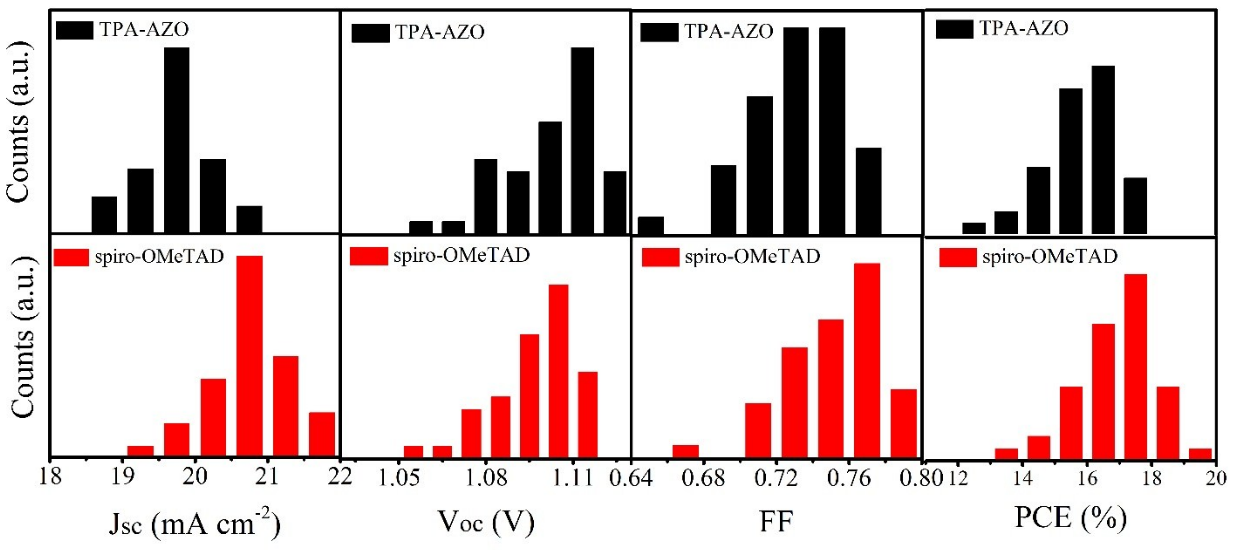

Figure S4. Histograms of the photovoltaic characteristics measured on 30 perovskite solar cells (PSCs) employing TPA-AZO and spiro-OMeTAD HTMs under AM1.5G illumination.

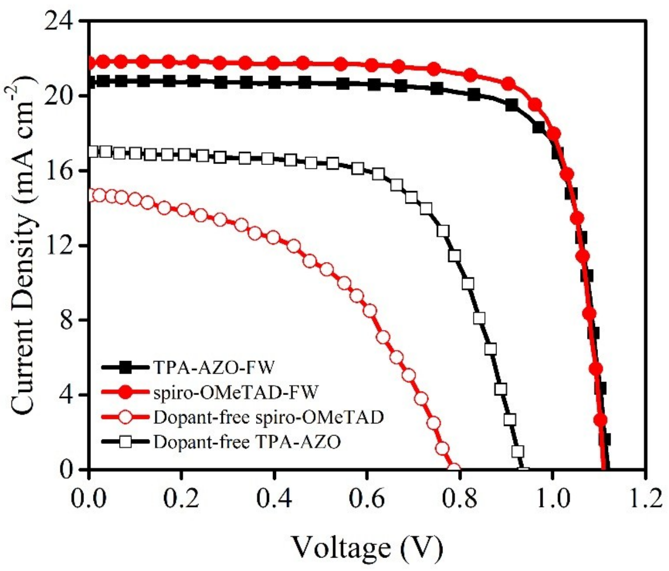

Figure S5. J–V curves of PSCs based on TPA-AZO and spiro-OMeTAD in their pristine form or doped with Li-TFSI and TBP, respectively.

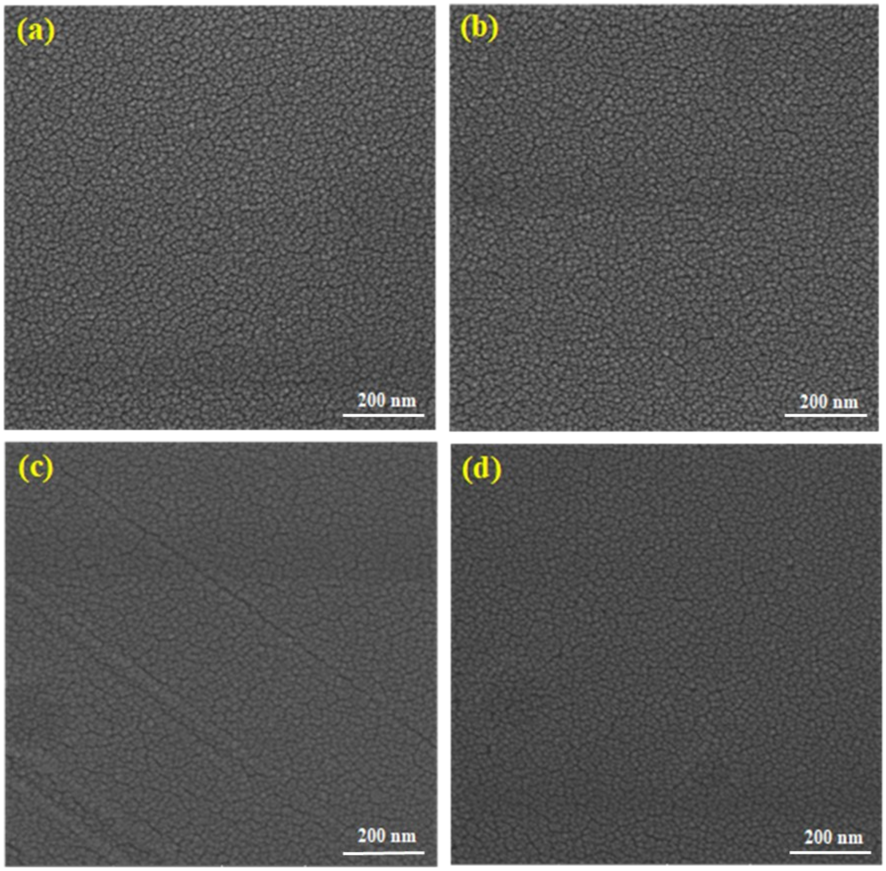

Figure S6. Top-view SEM images of (a) perovskite/dopant-free TPA-AZO; (b) perovskite/doped TPA-AZO;

(c) perovskite/dopant-free spiro-OMeTAD; (d) perovskite/doped spiro-OMeTAD. Scale bar is 200 nm.

Estimation of the synthesis cost of materials

Table S1 reports the synthesis cost of 1 g TPA-AZO, as estimated by the cost models adopted by Pablo et al.[1] and Osedach et al.[2] The price of the materials used was obtained from Merck, Sigma Aldrich, DeJong companies. The cost of 1 g of the TPA-AZO was compared with the cost of 1 g of the spiro-OMeTAD (**Table S2**), following previous approaches reported literature.[3] We also considered the cost of dopants used for TPA-AZO to estimate the overall cost of our optimized HTMs based on TPA-AZO and spiro-OMeTAD, as shown in **Table S3**.

Table S1. Quantities and costs of the materials used for the synthesis of 1 g of TPA-AZO.

	Chemical name	Weight reagent (g/g)	Weight solvent (g/g)	Weight workup (g/g)	Price of chemical ($/kg)	Material cost ($/g product)	Cost per step ($/step)
Product Step 1 (Yield: 65%, 1.76 g)	triphenylamine	1			2.14	1.926	5.80
	Br_2	1			1	0.9	
	$CHCl_3$		23		66	0.91	
	$CHCl_3$			52	66	2.06	
Product Step 2 (Yield: 45%, 0.66 g)	N-Phenyl-4-(phenyldiazenyl)aniline	0.982			12000	5.16	16.96
	1,10-phenantholine	0.1			4000	0.4	
	Cs_2CO_3	2			1000	0.9	
	CuI	0.05			439	0.022	
	Anhydrous $MgSO_4$	1			66	0.066	
	Silica	20			130	2.6	
	Anhydrous DMF		5		80	0.1	
	$CHCl_3$		45		66	1.98	
	CH_2Cl_2		76		13	0.74	
	n-Hexane		66		50	5	
Total							22.76

Table S2. Quantities and cost of the materials used for the synthesis of 1 g of spiro-OMeTAD. Reproduced from ref.

4 with permission from the Royal Society of Chemistry, copyright 2016.

**Chemical name**	**Weight reagent (g/g)**	**Weight solvent (g/g)**	**Weight workup (g/g)**	**Price of chemical ($/kg)**	**Material cost ($/g product)**	**Cost per step ($/step)**
2,2',7,7'-tetrabromo-9,9'-spirobi[9H-fluorene]	1.15			95900.00	110.29	
4,4'-dimethoxydiphenylamine	1.87			54900.00	102.66	
t-BuONa	1.04			307.00	0.32	
$Pd_2(dba)_3$	0.067			14900.00	1.00	
Toluene		12		69.48	0.83	
Ethyl acetate			135	80.16	10.82	273.62
NaCl (brine)	2		1	50.70	0.05	
$MgSO_4$	0.05		1	144.20	0.14	
Ethyl acetate	1		120	80.16	9.62	
n-Hexane			176	117.91	20.75	
Silica gel 60	20		263	62.20	16.36	
Total						273.62

Table S3 Costs of pristine HTMs (TA-AZO and spiro-OMeTAD) and quantities and costs of the dopants used for the 1 g of HTM.

Compound	Cost per g ($/g)	Cost per g of LiTFSI ($)	Cost per g of TBP ($)	Amount required of LiTFSI + TBP per 1g of HTM (g)	Total cost of HTM and LiTFSI per 1 g of HTM ($)
TPA-AZO	22.76	5.04 (Aldrich Co.)	6.28 (Aldrich Co.)	0.124 + 0.367	25.68
spiro-OMeTAD	273.62	5.04 (Aldrich Co.)	6.28 (Aldrich Co.)	0.124 + 0.367	276.54

Table S4. Examples of organic HTMs reported in relevant literatures, together with: the catalysts used for their synthesis; the synthesis cost for 1 g of materials; the photovoltaic characteristics of the corresponding PSCs under AM1.5G illumination.

**No.**	**HTM**	**Catalyst used for synthesis**	**Cost per 1 g of HTM ($/g)**	**Jsc ($mA\ cm^{-2}$)**	**Voc (V)**	**FF**	**η (%)**	**Ref.**
1	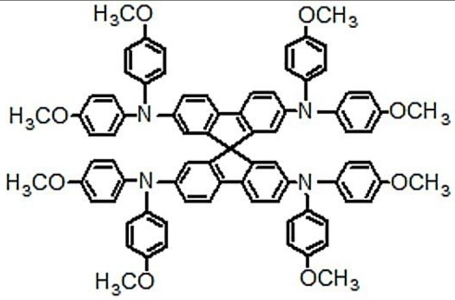	t-BuONa<br>$Pd2(dba)_3$<br>$P(t\text{-}Bu)_3$	273.62	20.7	1.00	0.71	14.9	5
2	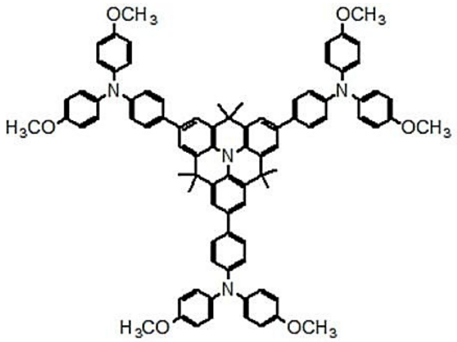	$Pd(PPh_3)_4$<br>K_2CO_3	842.08	20.98	0.97	0.67	13.63	6
3	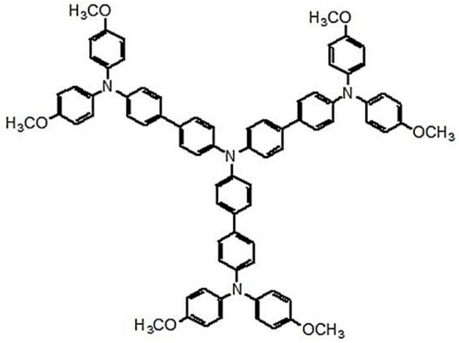	$Pd(PPh_3)_4$<br>K_2CO_3	420.22	20.88	0.95	0.62	12.31	6
4	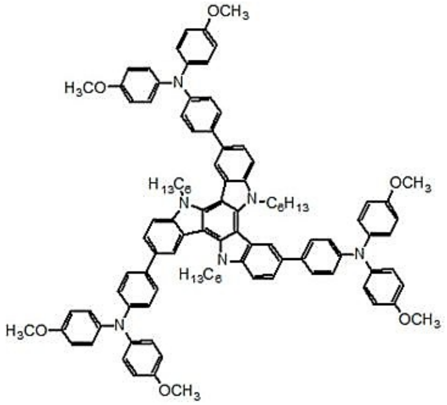	$Pd(PPh_3)_4$<br>K_2CO_3	695.87	21.21	1.09	0.78	18.36	7

5	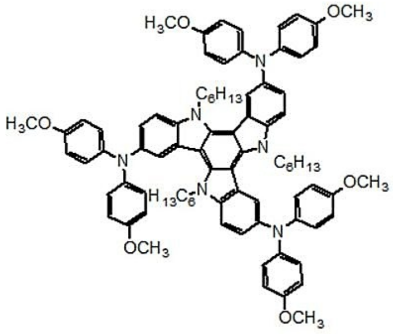	t-BuONa<br>$Pd(OAc)_2$<br>$P(t\text{-}Bu)_3$	245.84	20.6	0.88	0.63	11.54	7
6	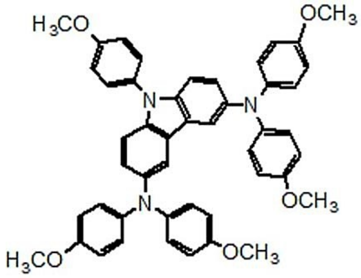	t-BuONa<br>$P(t\text{-}Bu)_3$<br>$Pd_2(dba)_3$	148.57	20.4	1.13	0.68	15.8	8
7	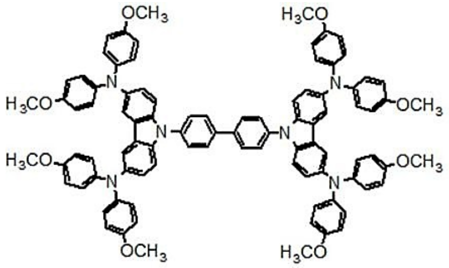	CuI<br>1,10-phenanthroline<br>K_2CO_3	216.46	23.2	1.02	0.79	18.6	8
8	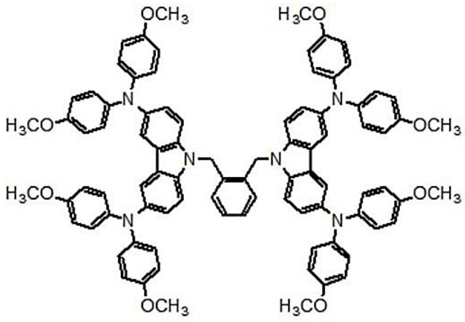	t-BuONa<br>$Pd(OAc)_2$<br>$[(t\text{-}Bu)_3PH]BF_4$	168.42	21.0	0.92	0.67	12.92	9
9	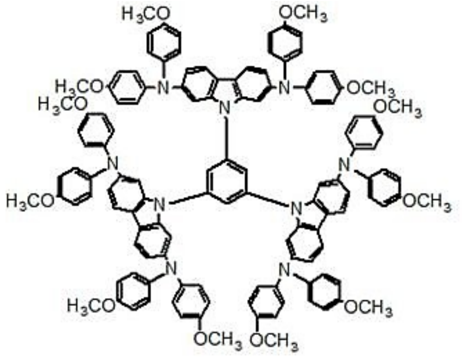	$Pd(OAc)_2$<br>Tri-t-butylphosphine<br>t-BuONa	434.12	20.28	1.02	0.71	14.79	10

10	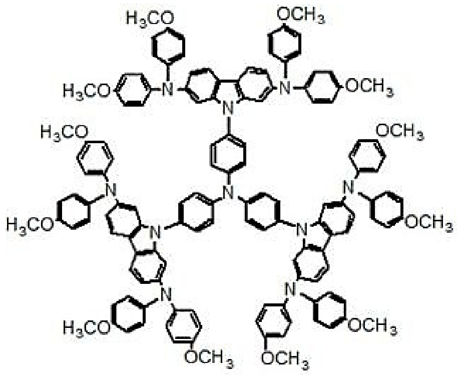	$Pd(OAc)_2$<br>Tri-t-butylphosphine<br>t-BuONa	450.13	20.35	0.99	0.69	13.86	[10]
11	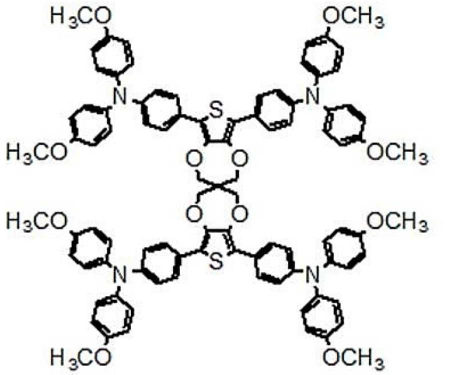	K_2CO_3<br>$Pd(PPh_3)_4$	579.16	17.63	1.02	0.73	13.44	[11]
12	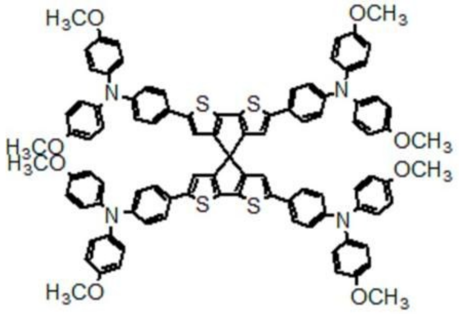	K_2CO_3<br>$Pd(PPh_3)_4$	633.88	13.8	0.98	0.76	10.3	[12]
13	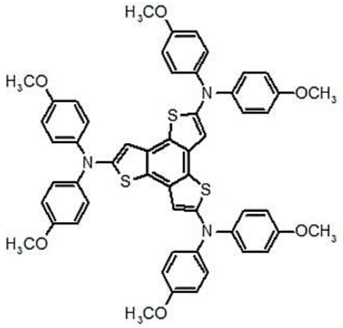	$Pd_2(dba)_3$<br>X-Phos<br>t-BuONa	376.30	20.4	1.04	0.72	16.0	[13]

14	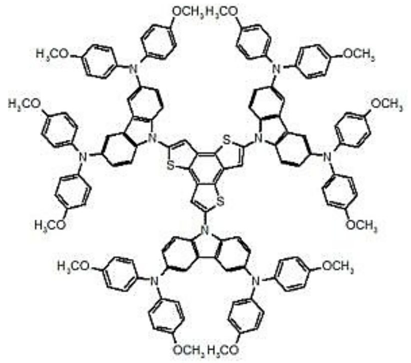	$Pd_2(dba)_3$<br>X-Phos<br>t-BuONa	591.57	20.6	1.09	0.77	17.0	[13]
15	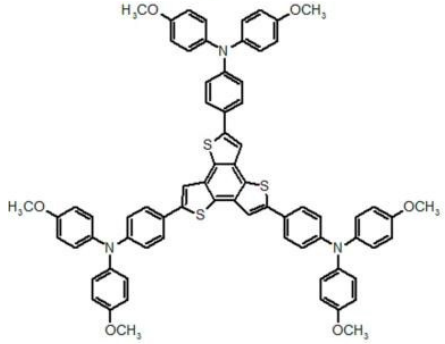	$Pd(PPh_3)_4$<br>K_3PO_4<br>NH_4Cl	800.49	21.9	1.07	0.77	18.2	[13]
16	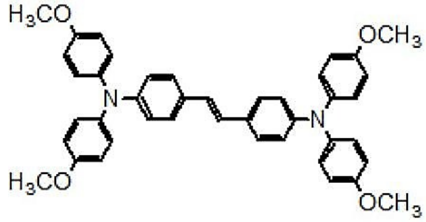	Zn<br>$TiCl_4$	101.34	18.2	1.03	0.61	11.4	[14]
17	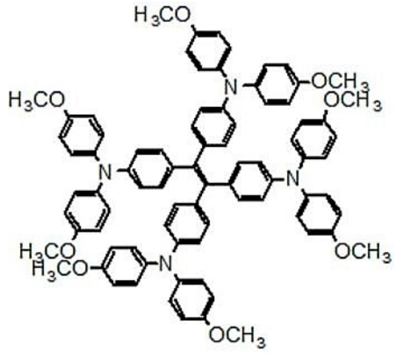	Zn<br>$TiCl_4$	52.59	21.2	0.92	0.67	13.1	[14]
18	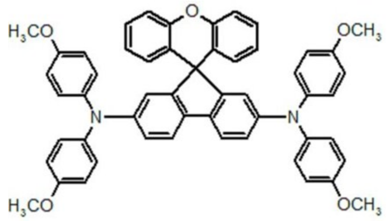	t-BuONa<br>$P(t\text{-}Bu)_3$<br>$Pd(OAc)_2$	112.23	23.4	1.13	0.73	19.8	[15]

19	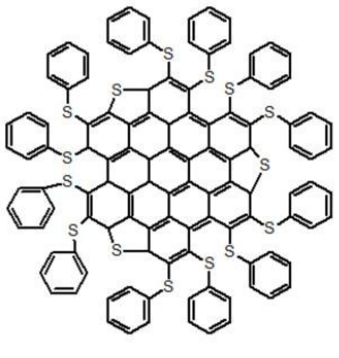	AlC_3<br>$FeCl_3$	367.55	20.6	0.95	0.66	12.8	[16]
20	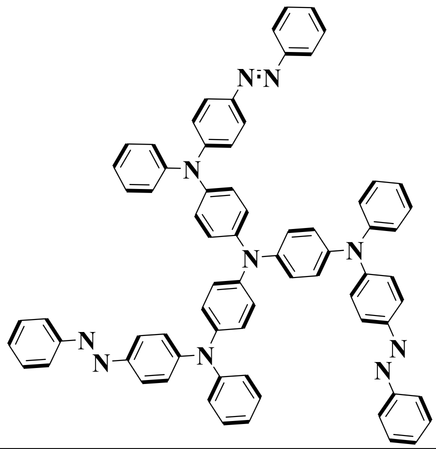	CuI<br>Cs_2CO_3<br>1,10-phenanthroline	22.76	17.01	0.94	0.63	17.8<br>6	This Work

Table S5. Figures of merit of PSCs based on various dopant-free HTMs under AM1.5G illumination, as reported in relevant literature.

**HTM**	**Jsc (mA cm^{-2})**	**Voc (V)**	**FF**	**η (%)**	**Ref.**
TPA-AZO	17.01	0.94	63	10.07	This Work
CuSCN	19.7	1.02	0.62	12.4	17
NiO	14.2	0.79	0.65	7.3	18
Cu_2O	15.8	0.96	0.59	8.93	19
Cu_2ZnSnS_4	20.54	1.06	0.59	12.75	20
$CuInS_2$/ZnS	18.6	0.92	0.49	8.4	21
CuMePc	16.9	0.70	0.40	5.2	22
$CuGaO_2$	21.7	1.11	0.77	18.5	23
SubPc	21.3	0.67	0.46	6.6	24
TiOPc	12.6	0.73	0.53	5.1	25
P3HT	24.3	0.98	0.57	13.6	26
PF8-TAA	6.1	1.40	0.79	6.7	27
PIF8-TAA	19.0	1.04	0.46	9.1	27
PANI	14.5	0.78	0.65	7.3	28
PCBTDPP	13.9	0.83	0.48	5.6	29
Conjugated D-A copolymer (P)	12.0	0.84	0.66	6.6	30
PTB-BO	14.4	0.83	0.62	7.4	31
PTB-DCB21	15.4	0.89	0.64	8.7	31
PDPP3T	20.5	0.98	61.2	12.3	32

References

1. M. Petrus, T. Bein, T. Dingemans and P. Docampo, *J. Mater. Chem. A*, 2015, **3**, 12159.
2. T. P. Osedach, T. L. Andrew and V. Bulović, *Energy Environ. Sci.*, 2013, **6**, 711.
3. (a) B. Xu, D. Bi, Y. Hua, P. Liu, M. Cheng, M. Grätzel, L. Kloo, A. Hagfeldt and L. Sun, *Energy Environ. Sci.*, 2016, **9**, 873; (b) M. Saliba, S. Orlandi, T. Matsui, S. Aghazada, M. Cavazzini, J.-P. Correa-Baena, P. Gao, R. Scopelliti, E. Mosconi and K.-H. Dahmen, *Nat. Energy*, 2016, **1**, 15017.
4. C. H. Teh, R. Daik, E. L. Lim, C. C. Yap, M. A. Ibrahim, N. A. Ludin, K. Sopian and M. A. M. Teridi, *J. Mater. Chem. A*, 2016, **4**, 15788.
5. N. J. Jeon, H. G. Lee, Y. C. Kim, J. Seo, J. H. Noh, J. Lee and S. I. Seok, *J. Am. Chem. Soc.*, 2014, **136**, 7837.
6. H. Choi, S. Paek, N. Lim, Y. H. Lee, M. K. Nazeeruddin and J. Ko, *Chem. -Eur. J.*, 2014, **20**, 10894.
7. K. Rakstys, A. Abate, M. I. Dar, P. Gao, V. Jankauskas, G. n. Jacopin, E. Kamarauskas, S. Kazim, S. Ahmad and M. Grätzel, *J. Am. Chem. Soc.*, 2015, **137**, 16172.
8. B. Xu, E. Sheibani, P. Liu, J. Zhang, H. Tian, N. Vlachopoulos, G. Boschloo, L. Kloo, A. Hagfeldt and L. Sun, *Adv. Mater.*, 2014, **26**, 6629.
9. P. Gratia, A. Magomedov, T. Malinauskas, M. Daskeviciene, A. Abate, S. Ahmad, M. Grätzel, V. Getautis and M. K. Nazeeruddin, *Angew. Chem. Int. Ed.*, 2015, **54**, 11409.
10. S. Do Sung, M. S. Kang, I. T. Choi, H. M. Kim, H. Kim, M. Hong, H. K. Kim and W. I. Lee, *Chem. Commun.*, 2014, **50**, 14161.
11. P. Ganesan, K. Fu, P. Gao, I. Raabe, K. Schenk, R. Scopelliti, J. Luo, L. H. Wong, M. Grätzel and M. K. Nazeeruddin, *Energy Environ. Sci.*, 2015, **8**, 1986.
12. M. Franckevičius, A. Mishra, F. Kreuzer, J. Luo, S. M. Zakeeruddin and M. Grätzel, *Mater. Horiz.*, 2015, **2**, 613.
13. A. Molina-Ontoria, I. Zimmermann, I. Garcia-Benito, P. Gratia, C. Roldán-Carmona, S. Aghazada, M. Graetzel, M. K. Nazeeruddin and N. Martín, *Angew. Chem. Int. Ed.*, 2016, **55**, 6270.
14. H. Choi, K. Do, S. Park, J. S. Yu and J. Ko, *Chem. -Eur. J.*, 2015, **21**, 15919.
15. D. Bi, B. Xu, P. Gao, L. Sun, M. Grätzel and A. Hagfeldt, *Nano Energy*, 2016, **23**, 138.
16. J. Cao, Y.-M. Liu, X. Jing, J. Yin, J. Li, B. Xu, Y.-Z. Tan and N. Zheng, *J. Am. Chem. Soc.*, 2015, **137**, 10914.
17. P. Qin, S. Tanaka, S. Ito, N. Tetreault, K. Manabe, H. Nishino, M. K. Nazeeruddin and M. Grätzel, *Nat. Commun.*, 2014, **5**, 3834.
18. A. S. Subbiah, A. Halder, S. Ghosh, N. Mahuli, G. Hodes and S. K. Sarkar, *J. Phys. Chem. Lett.*, 2014, **5**, 1748.
19. (a) M. I. Hossain, F. H. Alharbi and N. Tabet, *Sol. Energy*, 2015, **120**, 370; (b) B. A. Nejand, V. Ahmadi, S. Gharibzadeh and H. R. Shahverdi, *ChemSusChem*, 2016, **9**, 302.
20. Q. Wu, C. Xue, Y. Li, P. Zhou, W. Liu, J. Zhu, S. Dai, C. Zhu and S. Yang, *ACS Appl. Mater. Interfaces*, 2015, **7**, 28466.
21. M. Lv, J. Zhu, Y. Huang, Y. Li, Z. Shao, Y. Xu and S. Dai, *ACS Appl. Mater. Interfaces*, 2015, **7**, 17482.
22. G. Sfyri, C. V. Kumar, Y.-L. Wang, Z.-X. Xu, C. Krontiras and P. Lianos, *Appl. Surf. Sci.*, 2016, **360**, 767.
23. H. Zhang, H. Wang, W. Chen and A. K. Y. Jen, *Adv. Mater.*, 2017, **29**, 1604984.
24. G. Sfyri, C. V. Kumar, G. Sabapathi, L. Giribabu, K. S. Andrikopoulos, E. Stathatos and P. Lianos,

RSC Adv., 2015, **5**, 69813.
25. M. Sun, S. Wang, Y. Xiao, Z. Song and X. Li, *J. Energy Chem.*, 2015, **24**, 756.
26. H. Lu, Y. Ma, B. Gu, W. Tian and L. Li, *J. Mater. Chem. A*, 2015, **3**, 16445.
27. S. Ryu, J. H. Noh, N. J. Jeon, Y. C. Kim, W. S. Yang, J. Seo and S. I. Seok, *Energy Environ. Sci.*, 2014, **7**, 2614.
28. Y. Xiao, G. Han, Y. Chang, H. Zhou, M. Li and Y. Li, *J. Power Sources*, 2014, **267**, 1.
29. B. Cai, Y. Xing, Z. Yang, W.-H. Zhang and J. Qiu, *Energy Environ. Sci.*, 2013, **6**, 1480.
30. P. Nagarjuna, K. Narayanaswamy, T. Swetha, G. H. Rao, S. P. Singh and G. Sharma, *Electrochim. Acta*, 2015, **151**, 21.
31. J. W. Lee, S. Park, M. J. Ko, H. J. Son and N. G. Park, *ChemPhysChem*, 2014, **15**, 2595.
32. A. Dubey, N. Adhikari, S. Venkatesan, S. Gu, D. Khatiwada, Q. Wang, L. Mohammad, M. Kumar and Q. Qiao, *Sol. Energy Mater. Sol. Cells*, 2016, **145**, 193.
